# *VPS13D* mutations affect mitochondrial homeostasis and locomotion in *Caenorhabditis elegans*

**DOI:** 10.1093/g3journal/jkaf023

**Published:** 2025-02-17

**Authors:** Xiaomeng Yin, Ruoxi Wang, Andrea Thackeray, Eric H Baehrecke, Mark J Alkema

**Affiliations:** Department of Neurobiology, University of Massachusetts Chan Medical School, University of Massachusetts, Worcester, MA 01605, USA; Department of Molecular, Cell and Cancer Biology, University of Massachusetts Chan Medical School, University of Massachusetts, Worcester, MA 01605, USA; School of Life Sciences, Southern University of Science and Technology, Shenzhen 518055, China; Department of Molecular, Cell and Cancer Biology, University of Massachusetts Chan Medical School, University of Massachusetts, Worcester, MA 01605, USA; School of Life Sciences, Southern University of Science and Technology, Shenzhen 518055, China; Department of Neurobiology, University of Massachusetts Chan Medical School, University of Massachusetts, Worcester, MA 01605, USA; Department of Molecular, Cell and Cancer Biology, University of Massachusetts Chan Medical School, University of Massachusetts, Worcester, MA 01605, USA; Department of Neurobiology, University of Massachusetts Chan Medical School, University of Massachusetts, Worcester, MA 01605, USA

**Keywords:** *Caenorhabditis elegans*, *VPS13D*, *vps-13D*, mitochondrial homeostasis, WormBase

## Abstract

Mitochondria control cellular metabolism, serve as hubs for signaling and organelle communication, and are important for the health and survival of cells. *VPS13D* encodes a cytoplasmic lipid transfer protein that regulates mitochondrial morphology, mitochondria and endoplasmic reticulum contact, and quality control of mitochondria. *VPS13D* mutations have been reported in patients displaying ataxic and spastic gait disorders with variable age of onset. Here, we used CRISPR/Cas9 gene editing to create *VPS13D*-related spinocerebellar ataxia-4 missense mutations and C-terminal deletion in *VPS13D*'s ortholog *vps-13D* in *Caenorhabditis elegans*. Consistent with SCAR4 patient movement disorders and mitochondrial dysfunction, *vps-13D* mutant worms exhibit locomotion defects and abnormal mitochondrial morphology. Importantly, animals with a *vps-13D* deletion or a N3017I missense mutation exhibited an increase in mitochondrial unfolded protein response. The cellular and behavioral changes caused by *VPS13D* mutations in *C. elegans* advance the development of animal models that are needed to study SCAR4 pathogenesis.

## Introduction

Mitochondria are dynamic organelles that play an important role in cellular bioenergetics and viability. The mitochondrial network undergoes continuous fusion and fission to maintain its health. In addition, mitochondria form dynamic contacts with other organelles, especially the endoplasmic reticulum, to participate in various biological processes. Damaged mitochondria are selectively cleared by autophagy, and alterations in mitochondrial removal by autophagy have been associated with neurological disorders, including Alzheimer's disease, Parkinson's disease, Huntington's disease, and amyotrophic lateral sclerosis (Collier *et al*. 2023).

Vacuolar protein sorting-associated protein 13D, encoded by the *VPS13D* gene, is a key regulator of mitochondrial processes, including mitochondrial clearance, mitochondrial morphology, and interorganelle contacts (Anding *et al*. 2018; Guillén-Samander *et al*. 2021; Shen, Fortier, Zhao, *et al*. 2021). VPS13D is a member of the conserved VPS13 protein family, which facilitates membrane contacts and transports lipids between organelles (Leonzino *et al*. 2021). Unlike the other 3 human VPS13 proteins (VPS13A–C), VPS13D possesses a unique ubiquitin-associated (UBA) domain that has been shown to interact with K63 ubiquitin chains and participate in mitochondrial health (Anding *et al*. 2018; Leonzino *et al*. 2021). Mutations in the *VPS13A*–*C* genes have been linked to specific neurological disorders, including chorea-acanthocytosis, Cohen’s syndrome, and early-onset Parkinson's disease. Importantly, biallelic pathogenic variants in the *VPS13D* gene have also been associated with spastic ataxia and spastic paraplegia (Gauthier *et al*. 2018; Seong *et al*. 2018).

VPS13D movement disorder, also referred to as autosomal recessive spinocerebellar ataxia-4 (SCAR4; Swartz *et al*. 2002; Seong *et al*. 2018), manifests as a hyperkinetic movement disorder characterized by dystonia, chorea, and/or ataxia. This disorder is often accompanied with varying degrees of developmental delay and cognitive impairment (Meijer 2019). In the majority of the reported 42 cases, individuals have a combination of a loss-of-function variant and a seemingly milder variant, which can be either a missense mutation or a milder splicing variant (Gauthier *et al*. 2018; Seong *et al*. 2018; Koh *et al*. 2020; Lee *et al*. 2020; Petry-Schmelzer *et al*. 2021; Wang Yu *et al*. 2021; Durand *et al*. 2022; Huang and Fan 2022; Öztop-Çakmak *et al*. 2022; Baker *et al*. 2023; Kistol *et al*. 2023, 2024; Nordli and Galan 2023; Pauly *et al*. 2023; Algahtani *et al*. 2024; Harada *et al*. 2024; Sultan *et al*. 2024). Both *VPS13D*-deficient human and *Drosophila* cells exhibit notable abnormalities in mitochondrial morphology and clearance (Anding *et al*. 2018; Seong *et al*. 2018). In addition, a genetic screen for altered mitochondria in *Caenorhabditis elegans* identified *C25H3.11* (*vps-13D*) (Rolland and Conradt 2022). Recent studies have linked *VPS13D* to Leigh syndrome (Lee *et al*. 2020; Kistol *et al*. 2023) and the Parkinson's disease gene *PINK1* (Shen, Fortier, Wang, *et al*. 2021), suggesting that this gene has relevance to a broader spectrum of neurological diseases.

Strong loss-of-function *VPS13D* alleles are lethal in *Drosophila* (Anding *et al*. 2018) and mice (Skarnes *et al*. 2011), thus presenting a challenge to model this disease in an intact animal. In this study, we used CRISPR/Cas9 gene editing techniques to create SCAR4 patient-specific mutations in the *C. elegans* ortholog of the human *VPS13D* gene: *vps-13D*. We analyzed the effects of these SCAR4 patient-specific mutations in *vps-13D* on worm fecundity, behavior, and mitochondrial morphology. We find that *vps-13D* deletion mutants display maternal effect sterility and locomotion defects. *vps-13D* deletions and the N3017I missense mutation displayed disrupted mitochondrial morphology and resulted in different degrees of activated mitochondrial unfolded protein response (UPR^mt^). Furthermore, we identified a functional link between *vps-13D* and the mitochondria fusion regulator *fzo-1* in the same pathway to regulate mitochondrial dynamics and homeostasis. The combined biological changes caused by *VPS13D* mutations in *C. elegans* advance the development of a new animal model to study SCAR4 pathogenesis.

## Materials and methods

### *Caenorhabditis elegans* strains and GFP fusion construction

All *C. elegans* strains were maintained at 22°C on nematode growth medium (NGM) plates seeded with *Escherichia coli*OP50 bacteria. The wild-type strain was Bristol N2. Sterile mutant *vps-13D* strains were balanced with the *mIn1* balancer chromosome (Edgley and Riddle 2001). The *vps-13D* mutant strains were crossed with the following transgenes: *zcIs14* [*Pmyo-3::GFP* (*mit*)] (Benedetti *et al*. 2006) and *zcIs13* [*Phsp-6::GFP + lin-15* (*+*)] (Yoneda *et al*. 2004; Yang *et al*. 2022). A full list of strains used in this study is given in Supplementary Table 1. The *Pvps-13D*::*GFP* transcriptional reporter was generated using the following primers: F(5′-ACAGGATCCTCGCATACAATCACATCGTC-3′) and R(5′- ACAGGTACCTGTCCAGGAATTGTGGTATC-3′). These primers were used to amplify a 3.5-kb fragment corresponding to the upstream promoter sequence (including exon 1 and part of exon 2) of *vps-13D*. The PCR product was digested by *Bam*HI and *Kpn*I restriction enzymes and inserted into pPD95.75 vector. The *Pvps-13D*::*GFP* construct was microinjected at 50 ng/µl into temperature-sensitive *lin-15*(*n765ts*) mutant animals along with the *lin-15* (*+*) rescuing plasmid (pL15EK) at 80 ng/µl and pBSK DNA at 80 ng/µl. Transgenics were selected at 22°C based on a non-Muv (Multivulva) phenotype and GFP fluorescence.

### CRISPR/Cas9 design and gene editing

The Bristol N2 strain was used as the wild-type strain for all CRISPR/Cas9 editing of *vps-13D*. Site-specific crRNAs and a repair template donor ssODNs were manually selected and generated by IDT (Integrated DNA Technologies, Inc.) who also provided the tracrRNA. Injection mixtures were prepared following established protocols (Ghanta *et al*. 2021), and *rol-6* was used as a co-CRISPR selection marker. All crRNAs, ssODNs, and PCR screening sequences are reported in Supplementary Tables 2–4. Correct substitution or deletion sequences were confirmed via Sanger sequencing. Sequencing of C-terminal deletion *ΔC* (*zf197*) revealed a 1,218-bp deletion (LGII 5670774–5671991) with a short 20-bp insertion positioned between the 2 breakpoints. The CRISPR-designed strains underwent 4 rounds of outcrossing to the N2 strain.

### Brood size assay

Ten L4 animals of each genotype were picked and separated onto individual plates. Animals were transferred onto new plates every day until the cessation of egg laying. Plates were counted for the total number of eggs after removal of the parent animal.

### Larval development analysis

Five adult animals from each genotype were transferred to a new plate. After 2 h, adult animals were removed, and the eggs were left to develop for over 96 h. Two *vps-13D* deletion mutant strains were maintained as balanced heterozygotes using the *mIn1* [*dpy-10* (*e128*) *mIs14*] balancer. This balancer includes an integrated pharyngeal GFP reporter expressed in a semi-dominant manner. Consequently, the offspring segregate into wild type with a dim GFP signal, Dpy with a bright GFP signal (*mIn1* homozygotes), and non-GFP *vps-13D* deletion homozygotes. For the *vps-13D* deletion strains, only non-GFP homozygotes were evaluated at each developmental stage. The developmental timing was calculated as the proportion of larvae among the total number of hatched embryos that reached each specific developmental stage. The developmental stage of each individual was determined based on body size and stage-specific morphological features.

### Multi-Worm Tracker assay

Ten synchronized 1-day-old or 3-day-old (24 h or 3 days after the L4 stage) worms were placed on a thin lawn *E. coli*OP50 on a medium (5 cm) NGM plate. The plate was then placed in the Multi-Worm Tracker (MWT) [https://sourceforge.net/projects/mwt/, (Swierczek *et al*. 2011)] and recorded for 10 min. The MWT package includes real-time image analysis software and behavioral parameter measurement software, Choreography. Tracking and analysis were conducted following previous studies (Huang *et al*. 2019; Florman and Alkema 2022; Kang *et al*. 2024). The average speed of the population was measured over 5 min after a 5-min acclimation period. Experiments were analyzed using custom MATLAB (MathWorks, Inc.) scripts to interface with Choreography analysis program. The MATLAB scripts used in this study are available at https://github.com/jeremyflorman/Tracker_GUI. Analysis was limited to objects that had been tracked for a minimum of 20 s and had moved a minimum of 5 body lengths. For the analysis of single worm tracks, a plate containing a single 3-day-old animal was recorded for 10 min with images captured every 2 s. The sequential images from the final 5 min postacclimation were stitched and processed in ImageJ to generate a continuous worm movie track.

### Thrashing assay

Ten age-synchronized young adult animals were transferred to a fresh unseeded plate to remove residual bacteria. Individual animals were subsequently transferred to M9 buffer in a tiny plate. After 15-s acclimatization, the number of completed thrashes during 1 min was counted per animal using a hand counter.

### Gene expression knockdown via RNA-mediated interference treatment

*fzo-1* RNA-mediated interference (RNAi) bacterial clones, obtained from the Ahringer library (Kamath and Ahringer 2003), were selected by ampicillin (100 mg/ml) and tetracycline (12.5 mg/ml) and verified by DNA sequencing. The control L4440 or *fzo-1* RNAi bacteria grown at 37°C overnight in LB with ampicillin (100 mg/ml) were concentrated (4×) and seeded on RNAi NGM plates that contain 6 mM IPTG and 100 mg/ml ampicillin. Young adult P0 animals were placed on the RNAi plates and allowed to produce offspring. L4 larvae of F1 progeny were transferred to a new RNAi plate. After 2 generations of RNAi treatment, L4 larvae of F2 animals were analyzed.

### Imaging and fluorescence quantification

To assess the sterile phenotype, adult wild-type animals and *vps-13D* deletion mutant animals were placed on 2% agarose pads containing 60 mM sodium azide. Adult sterile animals were identified by the absence of embryos or oocytes in the uterus through DIC imaging using a Zeiss LSM 700 microscope. To explore the expression pattern of *vps-13D*, adult animals expressing a *Pvps-13D*::*GFP* reporter were mounted on 2% agarose pads containing 60 mM sodium azide. Images were captured using a Zeiss LSM 700 confocal microscope. To test the impact of *vps-13D* mutations on mitochondrial morphology, L4 animals expressing *zcIs14* transgene were mounted on 2% agarose pads containing 10 mM levamisole. Images were recorded using a Zeiss LSM 700 confocal microscope. For each genotype, mitochondrial morphology in body wall muscles in the middle of the worm body was scored for 10–15 animals in total. To test the effects of *vps-13D* mutations on *hsp-6* expression of *C. elegans*, 6 L4 animals expressing *Phsp-6*::*GFP* grown on *E. coli*OP50 were mounted together on 2% agarose pads containing 10 mM levamisole and 18–36 animals in total. The GFP images were acquired with an AxioImager Z1 microscope (Zeiss) at 10× magnification, and the maximum fluorescence intensity was quantified using ImageJ FIJI software.

### Mitochondrial morphology analysis

Mitochondrial images were segmented and quantified using the Mitochondrial Segmentation Network (MitoSegNet) toolbox, a deep learning-based tool that includes the MitoS segmentation tool and the MitoA analysis tool (Fischer *et al*. 2020). Documentation for the MitoS and MitoA tools is available at https://github.com/mitosegnet, and the MitoSegNet segmentation model, along with the MitoA analysis and MitoS segmentation tools (GPU/CPU versions) for Linux and Windows, can be accessed at https://zenodo.org/search?page=1&size=20&q=mitosegnet. Initially, 8-bit raw images were segmented using the pretrained mitochondria-specific “basic mode” (MitoSegNet model) in the MitoS tool (CPU version). Upon completion, the program generated a prediction folder containing the segmented images. The segmented images and corresponding raw images were then subjected to quantitative measurement using the MitoA tool, which evaluates morphological features for each object and generates summary statistics for all object features in each image. The major axis lengths were determined by measuring the lengths of the line segment connecting the 2 vertices of an ellipse fitted around an object in the MitoA tool.

### Statistical analysis

All statistical analysis and graph construction were performed using GraphPad Prism 8 software. The results are presented as SEM from at least 3 independent experiments. Statistical comparisons were made using ANOVA or Kruskal–Wallis *H* test with Dunnett's correction for multiple samples. Significance was determined when *P*-value < 0.05.

## Results

### *Caenorhabditis elegans vps-13D* encodes an ortholog of *VPS13D*

**Fig. 1.**
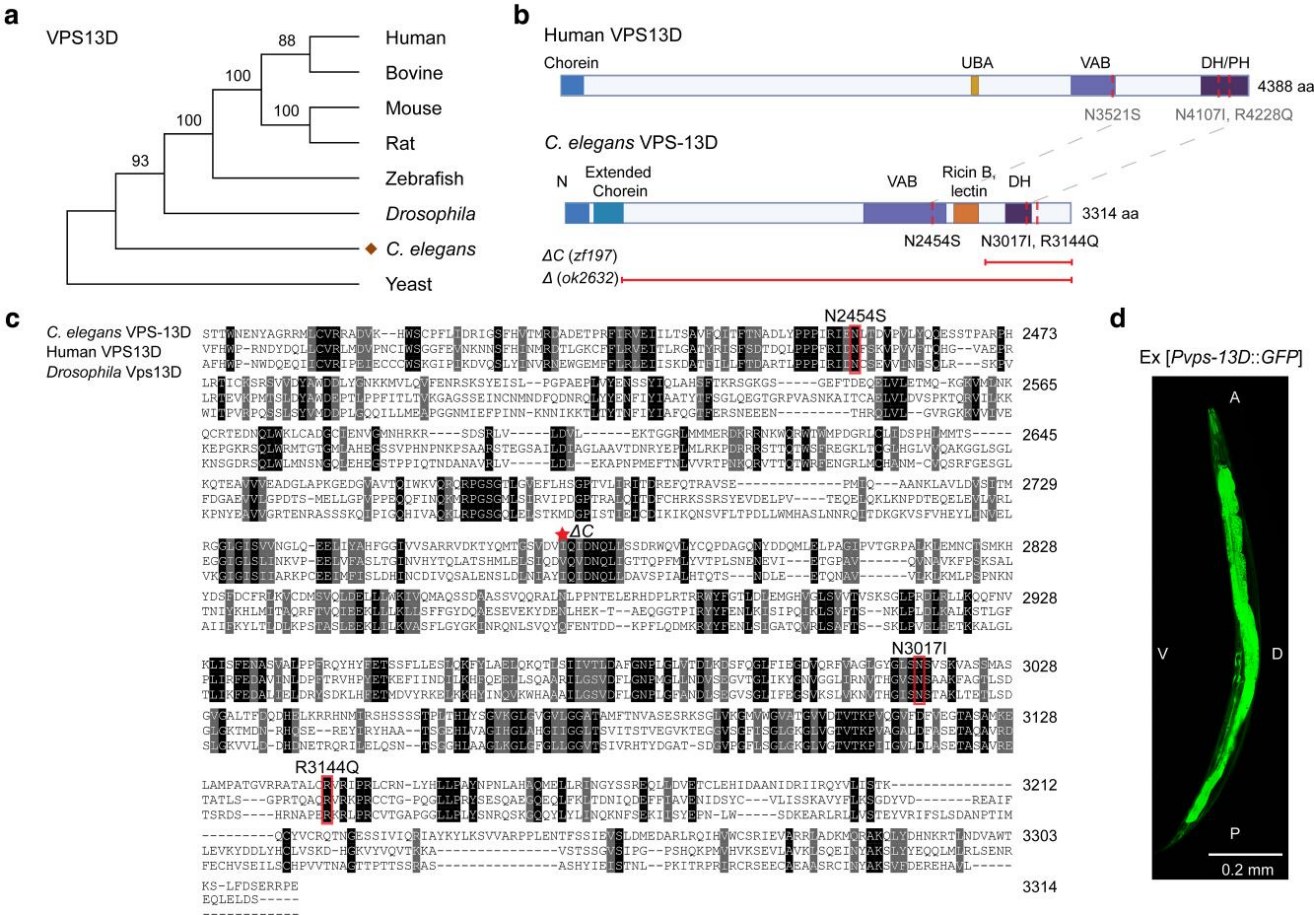
*VPS13D* ortholog *vps-13D* is conserved in *C. elegans*. a) Phylogenetic tree of VPS13D proteins. VPS13D protein sequences from various species were retrieved from UniProt. The evolutionary history was inferred using the neighbor-joining method. The percentage of replicate trees in which the associated taxa clustered together in the bootstrap test (1,000 replicates) is shown next to the branches. Evolutionary analyses were conducted in MEGA11. The species depicted from top to bottom are human (UniProt identifier: Q5THJ4), bovine (UniProt identifier: E1BIF6), mouse (UniProt identifier: B1ART2), rat (UniProt identifier: A0A8I6G572), zebrafish (UniProt identifier: A0A8M1QUR7), *Drosophila* (UniProt identifier: Q9VU08), *C. elegans* (labeled as brown diamond, UniProt identifier: A0A2K5ATR5), and yeast (UniProt identifier: Q07878). b) Human VPS13D protein topology [modified from (Guillén-Samander *et al*. 2021)] and predicted domains of the *C. elegans* VPS-13D protein with disease-related missense mutations labeled in dotted vertical lines and deletions annotated with horizontal lines. Domains and abbreviations are as follows: N, Chorein N-terminal domain; Chorein domain; Extended Chorein domain; UBA, ubiquitin-associated domain; VAB, Vps13 adaptor-binding/SHR-binding/WD40-like domain; ricin B-type lectin domain; DH, Dbl homology-like domain; and PH, pleckstrin homology domain. c) Sequence alignment of C-terminal region between *C. elegans* VPS-13D, human VPS13D, and fly Vps13D protein by Clustal Omega. The 3 missense mutations enrolled in this study are outlined, and the start residue of *vps-13D*(*ΔC*) deletion is labeled with asterisk. d) Representative fluorescence image of adult hermaphrodites expressing *Pvps-13D*::*GFP* under the 3,570-bp promoter (including exon 1 and part of exon 2). A, anterior; P, posterior; V, ventral side; D, dorsal side. The scale bar is 0.2 mm.

To explore the evolutionary relationships of the VPS13D proteins among various species, we first conducted a phylogenetic analysis. VPS13D proteins from different species formed a well-supported clade, indicating that they evolved from a common ancestor (Fig. 1a). The *C. elegansC25H3.11* gene encodes a protein that shares the most significant similarity with VPS13D. The predicted ricin B-type lectin domain-containing protein shares 36% similarity and 22% identity with the human VPS13D protein (Supplementary Table 5). Furthermore, the highest sequence similarity between the 2 proteins was observed in the C-terminal region, which contains the VPS13 adaptor-binding (VAB) domain (Bean *et al.* 2018) [also known as SHR-binding domain or WD40-like region (Kumar *et al.* 2018)] and a Dbl homology (DH)-like domain (InterPro) (Fig. 1b). Given that *C25H3.11* gene is the closest *C. elegans* ortholog of the human *VPS13D* gene, we hereafter refer to *C25H3.11* as the *C. elegansvps-13D* gene. In humans, 14 of 30 reported families carry at least 1 missense mutation in the C-terminal region following the UBA domain, and 5 of 8 reported homozygous missense mutations in patients are also located in this region (Gauthier *et al*. 2018; Seong *et al*. 2018; Koh *et al*. 2020; Lee *et al*. 2020; Petry-Schmelzer *et al*. 2021; Wang Yu *et al*. 2021; Durand *et al*. 2022; Huang and Fan 2022; Öztop-Çakmak *et al*. 2022; Baker *et al*. 2023; Kistol *et al*. 2023, 2024; Nordli and Galan 2023; Pauly *et al*. 2023; Algahtani *et al*. 2024; Harada *et al*. 2024; Sultan *et al*. 2024). Several of these mutation sites associated with human pathogenicity are highly conserved in the C-terminal region in *C. elegans* VPS13D protein (Fig. 1c).

We next focused on 3 conserved residues within the C-terminal region of VPS13D protein that are reported to be mutated in early-onset SCAR4 patients. We used CRISPR/Cas9 gene editing to create these variants in *vps-13D* [*N2454S(zf194)*, *N3017I*(*zf195)*, and *R3144Q(zf196)*] (Table 1). In addition, we generated a C-terminal deletion *ΔC*(*zf197*) that encompassed 1,218 bp, resulting in a truncation of the VPS13D protein (Fig. 1b). We also obtained a *vps-13D* deletion allele, *Δ*(*ok2632*), which contains a 1,740-bp deletion removing part of exons 3 and 4, resulting in a premature stop and thus most likely represents a null allele. To analyze the expression of *vps-13D*, we investigated its expression pattern by generating a transcriptional fusion of GFP reporter with 3.5-kb *vps-13D* upstream promoter fragment (including exon 1 and part of exon 2). Fluorescence from the *Pvps-13D*::*GFP* reporter was observed throughout the adult hermaphrodite, including intestine, hypodermis, muscle, glia, and neurons (Fig. 1d). Our data indicate that *vps-13D* is expressed in most cell types consistently with RNA expression datasets (Taylor *et al*. 2021).

**Table 1.** VPS13D pathogenic missense mutations in this study.

*Caenorhabditis elegans* mutations (*vps-13D* a.1)	VPS13D pathogenic mutations (NM_015378)	Clinical diagnosis of patients	Onset age
N2454S (*zf194*)	p.N3521S (c.10562A > G)	Spastic ataxia with mild intellectual disability (2 patients)	2 years/4–5 years
N3017I (*zf195*)	p.N4107I (c. 12320A > T)	Ataxia, neuropathy, developmental delay, seizures, and optic atrophy	1 year
R3144Q (*zf196*)	p.R4228Q (c.12683G > A)	Ataxia, muscle weakness, dystonia, mild intellectual disability	1–2 years

### *vps-13D* mutants exhibit impaired locomotion

**Fig. 2.**
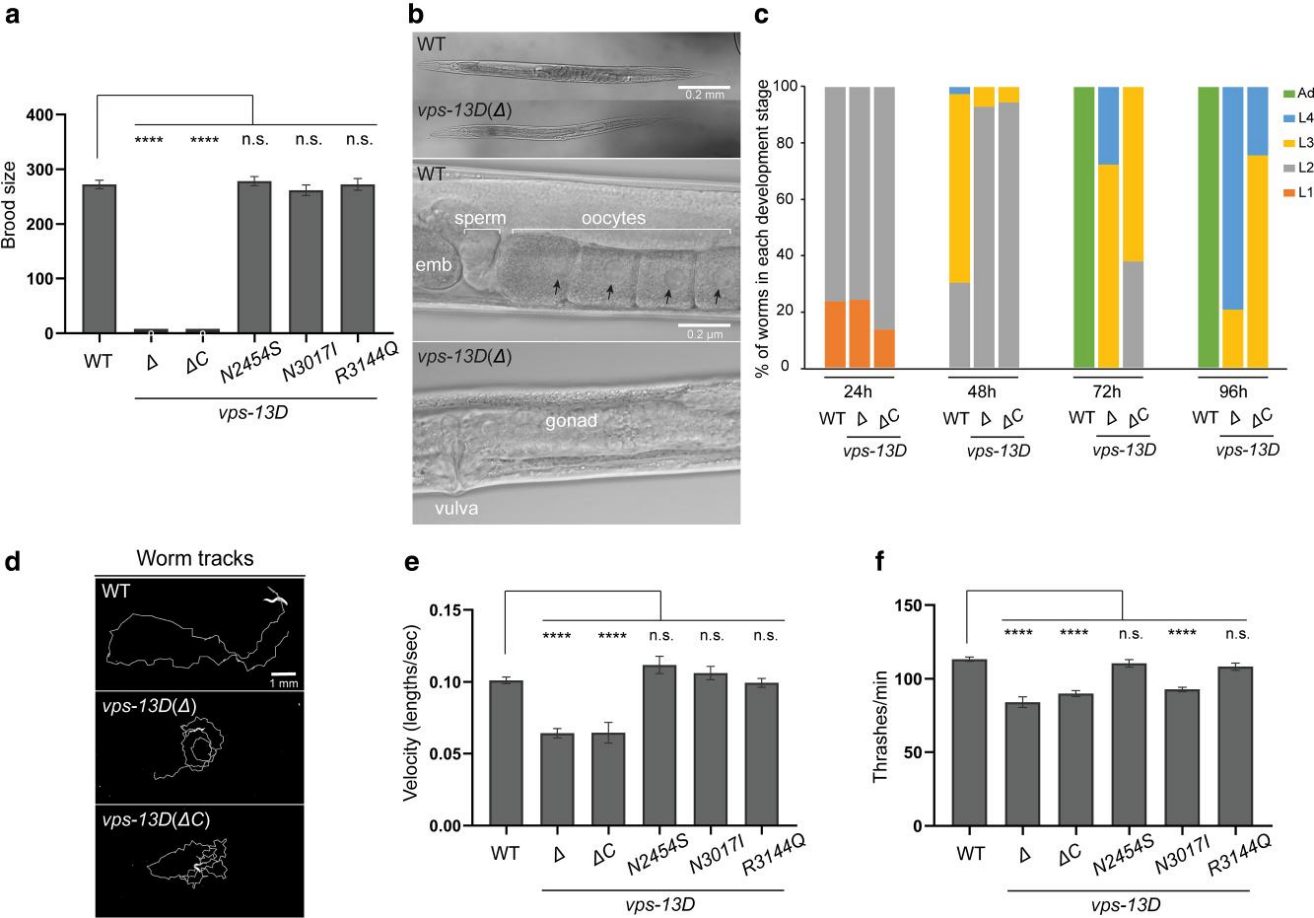
*vps-13D* deficiency affects worms’ fertility and locomotion. a) Total brood size (*n* = 30 broods) indicates the fertility ability of *C. elegans* to produce offspring in association with different *vps-13D* mutants. One-way ANOVA with Dunnett’s correction was used to compare the difference between wild-type and mutant animals. b) DIC images of whole worm and gonad in hermaphrodites of the wild-type and *vps-13D* (*Δ*) mutant strains. The scale bar in the whole worm image is 0.2 mm. The scale bar in the gonad image is 0.2 μm. Black arrows indicate oocyte nuclei. c) The percentage of worms at the different developmental stages was determined for wild-type, *vps-13D*(*Δ*), and *vps-13D*(*ΔC*) mutant animals after 96 h of growth from the embryonic stage at 22°C. d) Individual animal tracks of 3-day-old wild-type, *vps-13D*(*Δ*), and *vps-13D*(*ΔC*) mutant strains for the final 5-min recordings. The scale bar is 1 mm. e) Comparison of average velocity for 3-day-old wild-type and *vps-13D* animals from the final 5-min MWT recordings. The velocity has been normalized and calculated based on the worm body lengths of respective strains. The difference in average velocity between wild type and mutants was analyzed by One-way ANOVA with Dunnett's multiple comparison tests. f) Number of thrashes per minute in M9 liquid for 3-day-old wild-type and *vps-13D* mutant animals (*n* = 15–30). Differences between wild type and mutants were analyzed by one-way ANOVA with Dunnett’s correction for multiple comparisons. **** *p* < 0.0001; n.s., not significant.

Animals carrying either homozygous *vps-13D* deletion alleles [*Δ*(*ok2632*) or *ΔC*(*zf197*)] are viable. However, both homozygous *vps-13D*(*Δ*) and *vps-13D*(*ΔC*) animals display maternal effect sterility (Fig. 2a). Wild-type hermaphrodites produce oocytes that are fertilized in the spermatheca, resulting in the accumulation of developing embryos in the uterus. The germlines of either homozygous *vps-13D*(*Δ*) or *vps-13D*(*ΔC*) animals did not display visible oocytes or embryos (Fig. 2b). Due to their sterility, these 2 deletion strains were maintained as balanced heterozygotes but analyzed as homozygotes by selecting homozygous animals from a mixed population. In addition to fertility deficiencies, these 2 deletion mutants also exhibited a slower rate of development, taking 24–48 h longer than the wild type to reach the L4 stage (Fig. 2c). In contrast, 3 missense mutants *vps-13D(N2454S)*, (*N3017I*), and (*R3144Q*) did not exhibit developmental arrest or delay and were able to produce a similar brood size compared with the wild type (Fig. 2a).

Next, we examined whether *vps-13D* mutant strains possess motor defects. We conducted a population-level motility assay using an automated multiworm tracking system (MWT) (Swierczek *et al*. 2011). We tracked wild-type and *vps-13D* mutant strains for 5 min and extracted the behavioral dynamics of individual worms. One-day-old adult *vps-13D* mutants did not show any difference compared with the wild type (Supplementary Fig. 1). However, *vps-13D* deletion animals displayed a noticeable movement impairment compared with wild-type animals. The average locomotion rate of 3-day-old wild-type animals was 0.101 ± 0.005 body lengths/s, whereas either *vps-13D*(*Δ*) or *vps-13D*(*ΔC*) mutants displayed decreased locomotion rates of 0.064 ± 0.010 and 0.065 ± 0.012 body lengths/sec (Fig. 2d and e). The 3 *vps-13D* missense mutant strain locomotion rates were unaffected at day 3 of the adult stage (0.111 ± 0.009, 0.106 ± 0.010, and 0.099 ± 0.008 body lengths/s, respectively, Fig. 2e). Furthermore, we analyzed thrashing rates of single worm in liquid M9 medium. The average thrashing rate of 3-day-old wild-type animals was 113.2 ± 6.9 thrashes/min. Significantly, *vps-13D*(*Δ*) and *vps-13D*(*ΔC*) mutant strains showed a 20–26% decrease in thrashing rates compared with control wild-type animals (83.8 ± 11.0 and 89.8 ± 8.7 thrashes/min, respectively, Fig. 2f). Importantly, the thrashing rate of *vps-13D(N3017I)* mutant worms was 92.7 ± 7.1 thrashes/min, showing an 18% reduction compared with control animals (Fig. 2f), where *vps-13D(N2454S)* and *vps-13D(R3144Q)* mutants exhibited similar thrashing rates to the wild type (110.5 ± 11.0 and 108.3 ± 9.5 thrashes/min, respectively). Combined, these results indicate that the *C. elegansvps-13D* mutations can result in locomotion defects.

### *vps-13D* mutants possess altered mitochondrial morphology

**Fig. 3.**
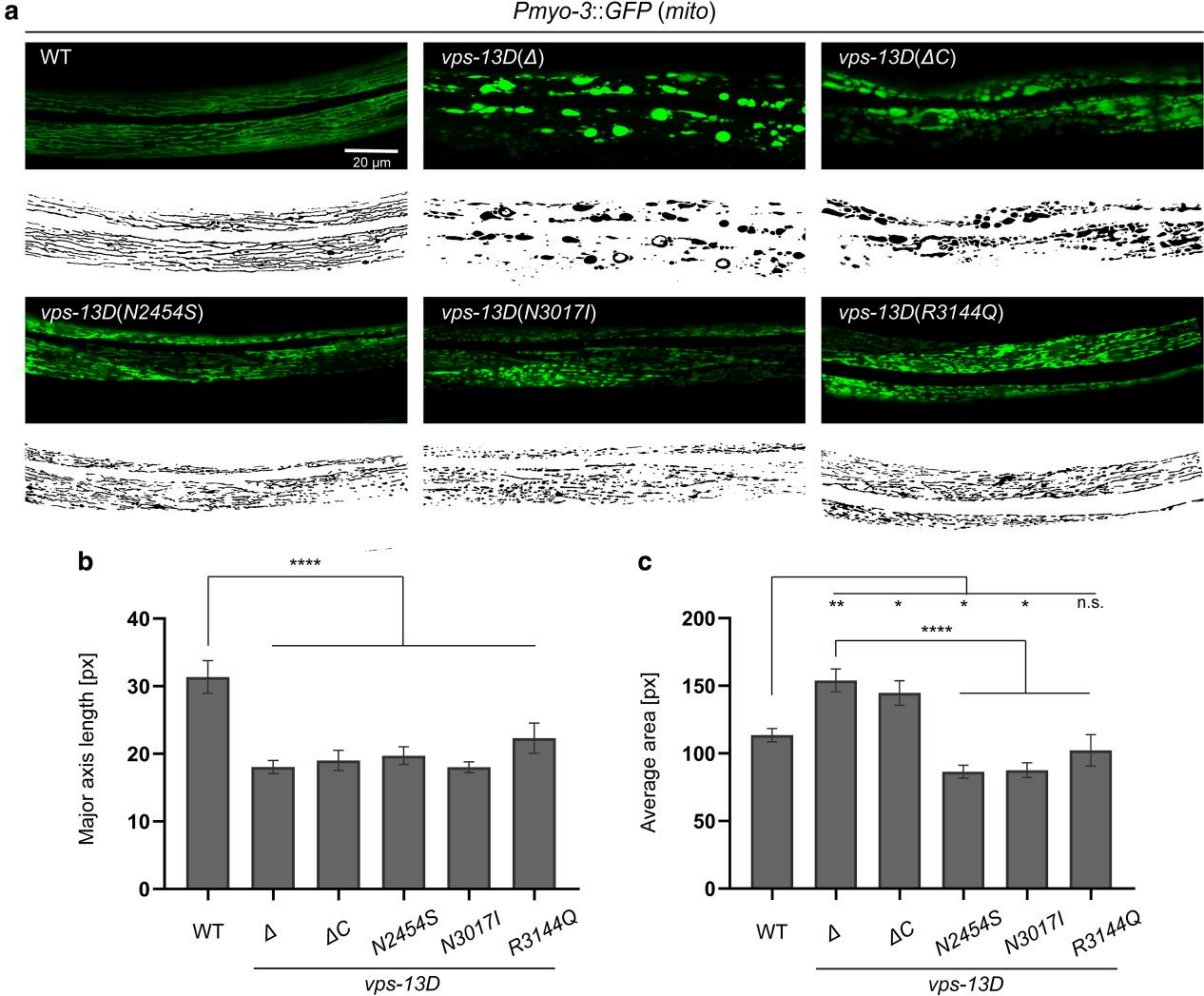
*vps-13D* regulates mitochondrial morphology in *C. elegans*. a) Mitochondrial morphology of the wild-type and *vps-13D* mutant animals. Original images are at the top, and MitoSegNet model segmentations are at the bottom. The scale bar is 20 μm. b) and c) Statistical analysis of mitochondrial morphology parameters. The major axis length (‘see *Materials and Methods*’) and average area were measured in segmented images of mitochondria from wild-type and *vps-13D* mutant animals (*n* = 15–25). One-way ANOVA with Dunnett’s correction was used to compare the difference between wild-type and mutant animals. Data are presented as mean ± SEM. *p < 0.05, **p < 0.01, ****p < 0.0001; n.s., not significant.

Dynamic changes in mitochondrial morphology are essential for mitochondrial health and homeostasis. Mitochondrial fusion and fission not only affect mitochondrial morphology but also regulate multiple mitochondrial biological processes, including mitochondrial quality control and clearance by mitophagy (Pickles *et al*. 2018). Previous studies indicate that VPS13D affects mitochondrial morphology and mitophagy in both human and *Drosophila* cells (Anding *et al*. 2018). To test whether *vps-13D* mutants exhibit a similar function to maintain mitochondrial morphology, we used a transgenic line *zcIs14* {[*myo-3::GFP* (*mit*)]} that labels mitochondria in the body wall muscles (Benedetti *et al*. 2006) and quantified the mitochondrial morphology using MitoSegNet (Fischer *et al*. 2020). Although morphologies between different mutants were variable and possibly reflected allele strength, mutant strains of *vps-13D* displayed changes in mitochondrial morphology at the L4 larval stage compared with the wild type (Fig. 3a). Specifically, all homozygous *vps-13D* mutant worms possessed shorter mitochondria compared with control wild-type worms, as determined by the major axis length (Fig. 3a and b). In wild-type worms, the major axis length was 31.4 ± 11.6 pixels. By contrast, *vps-13D*(*Δ*) and *vps-13D*(*ΔC*) mutant strains displayed significantly reduced major axis lengths of 18.1 ± 3.7 and 19.0 ± 5.4 pixels, respectively (Fig. 3b). Similarly, the major axis length was also reduced in the 3 *vps-13D* missense mutant strains, measuring 19.7 ± 5.7, 18.0 ± 3.9, and 22.3 ± 9.5 pixels, respectively (Fig. 3b). These data are consistent with a recent report (Rolland and Conradt 2022). In addition, both *vps-13D*(*Δ*) and *vps-13D*(*ΔC*) mutant strains demonstrated a notable increase in mitochondrial area measuring 154.0 ± 32.6 and 144.7 ± 30.2 pixels, respectively, compared with 113.5 ± 19.6 pixels in wild-type worms. By contrast, *vps-13D(N2454S)* and *vps-13D(N3017I)* mutant strains display slightly smaller mitochondrial areas of 86.5 ± 20.9 and 87.6 ± 26.5 pixels, respectively, where *vps-13D(R3144Q)* mutant is similar to the wild type (102.3 ± 49.4 pixels) (Fig. 3a and c). These results indicate that SCAR4 mutations in the *C. elegans VPS13D* ortholog *vps-13D* lead to alterations in mitochondrial morphology, supporting VPS-13D's role in maintaining mitochondrial structure.

### *vps-13D* disruption induces mitochondrial UPR^mt^

**Fig. 4.**
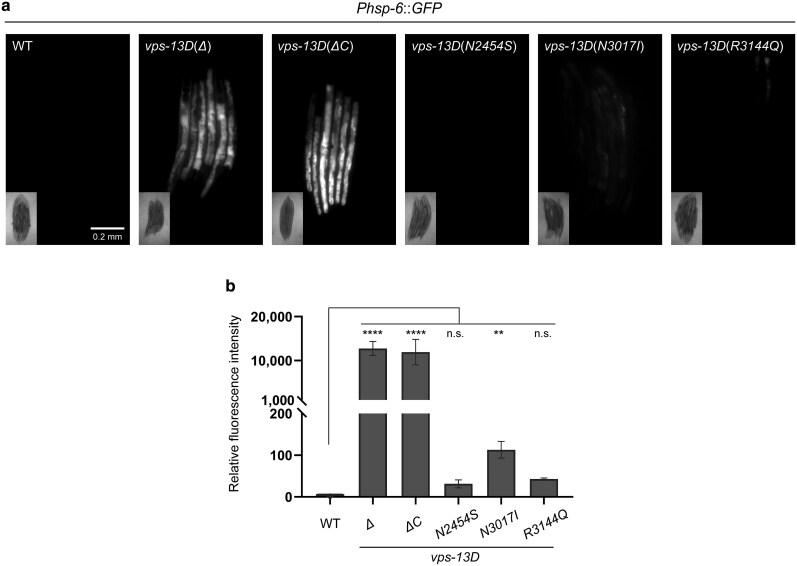
*vps-13D* dysfunction induces mitochondrial UPR. a) *Phsp-6*::*GFP* expression of wild-type and *vps-13D* mutant worms. The scale bar is 0.2 mm. b) Quantification of *Phsp-6*::*GFP* relative fluorescence intensity (*n* = 3–5 groups*6 animals). Kruskal–Wallis *H* test with Dunnett’s correction was used to compare the difference between wild-type and mutant animals. Data are presented as mean ± SEM. **P < 0.01, ****P < 0.0001; n.s., not significant.

Although previous assays have examined the effects of *VPS13D* mutations on mitochondrial morphology, their impact on mitochondrial homeostasis remains unclear (Anding *et al*. 2018). The mitochondrial UPR^mt^ is a crucial cellular pathway that protects mitochondria from multiple forms of cell stress (Shpilka and Haynes 2018). To test whether *vps-13D* mutants affect UPR^mt^, we used a transgene of the transcriptional reporter *Phsp-6::mtHSP70::GFP* to monitor UPR^mt^ in *C. elegans* (Yoneda *et al*. 2004; Yang *et al*. 2022). Interestingly, *vps-13D*(*Δ*) and *vps-13D*(*ΔC*) mutant strains exhibit a significant induction of the UPR^mt^ with high levels of *Phsp-6::mtHSP70::GFP* intensity compared with wild-type animals at L4 larval age (Fig. 4a and b). While the relative fluorescence intensity of *vps-13D(N2454S)* and *vps-13D(R3144Q)* missense mutants was not significantly different from wild-type, homozygous *vps-13D(N3017I)* mutant worms exhibited a slight increase in UPR^mt^ reporter expression compared with control worms (Fig. 4a and b). Combined, these results indicate that VPS13D plays a crucial role in maintaining mitochondrial homeostasis. Since the large deletion *Δ(ok2632)* allele and the C-terminal deletion allele *ΔC* (*zf197*) cause similar deficiencies in fertility, development, locomotion, as well as mitochondrial morphology, we cannot exclude that frameshift mutations near the C-terminus could compromise protein stability.

### *vps-13D* and *fzo-1/MFN2* function in a pathway to regulate mitochondrial homeostasis

**Fig. 5.**
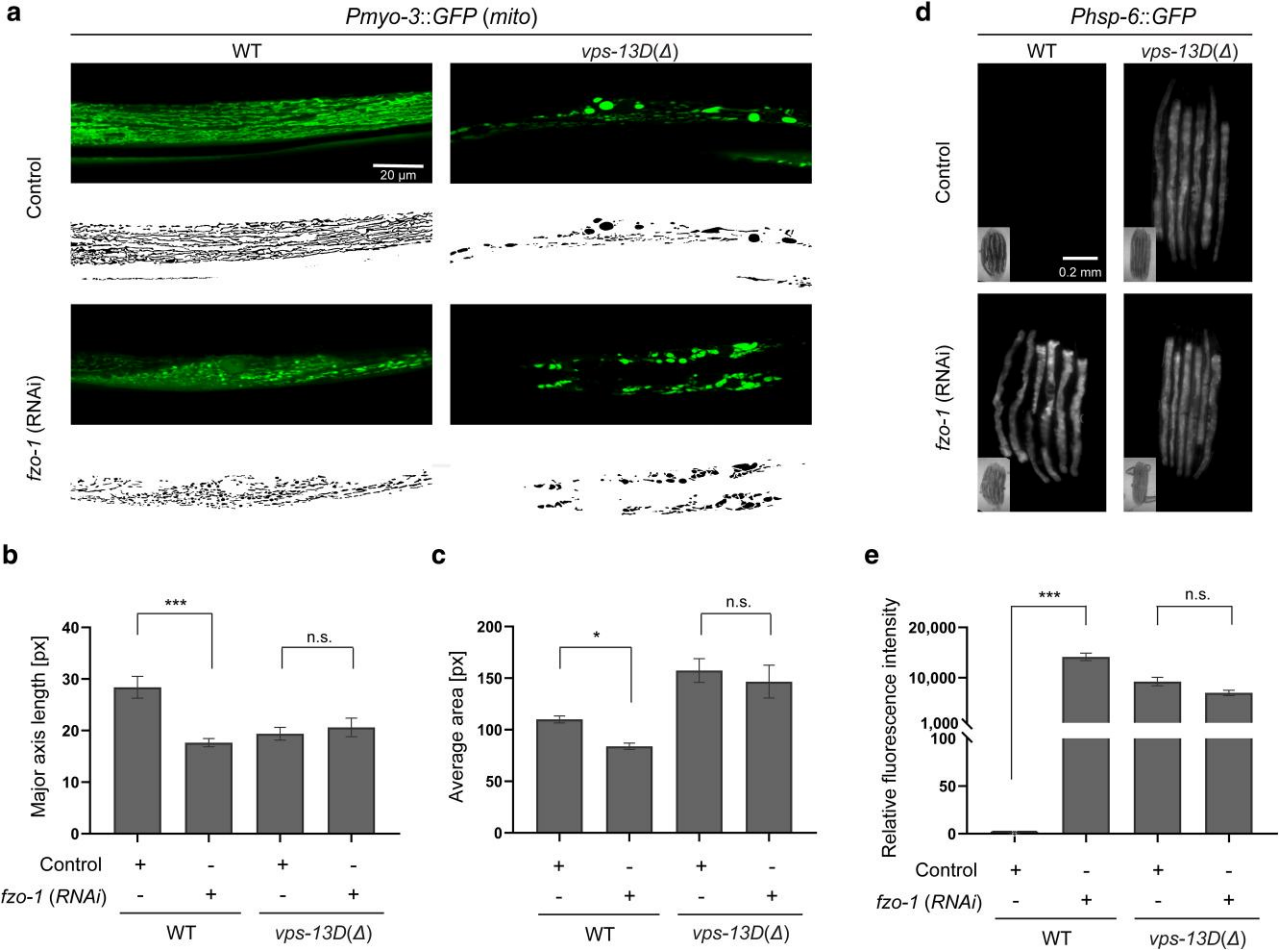
*vps-13D* and *fzo-1* regulate mitochondrial homeostasis in similar pathways. a) Mitochondrial morphology changes of wild-type and *vps-13D*(*Δ*) animals grown on either control mock or *fzo-1* (*RNAi*). Original images are at the top and MitoSegNet model segmentations are at the bottom. The scale bar is 20 μm. b) and c) Statistical analysis of mitochondrial morphology parameters. The major axis length (‘see *Materials and Methods*’) and average area were measured in segmented images of mitochondria from wild-type and *vps-13D*(*Δ*) animals grown on either control mock or *fzo-1* (*RNAi*) (*n* = 10–15). d) and e) *Phsp-6*::*GFP* expression of wild-type and *vps-13D*(*Δ*) animals grown on either control mock or *fzo-1* (*RNAi*) (*n* = 3–15 groups*6 animals). The scale bar is 0.2 mm. Kruskal–Wallis *H* test with Dunnett’s correction was used to compare the difference between wild-type and mutant animals. Data are presented as mean ± SEM. *p < 0.05, ***p < 0.001; n.s., not significant.

In *Drosophila*, the ortholog of *mitofusin-2 (MFN2)*, *Marf* acts downstream of Vps13D to regulate mitochondrial fusion (Shen, Fortier, Zhao, *et al*. 2021). The *C. elegans* MFN2 ortholog FZO-1 is also involved in mitochondrial fusion (Rolland *et al*. 2009). Since our results indicate that *vps-13D* loss promotes alteration of mitochondrial morphology (Fig. 3), we examined the potential relationship between VPS-13D and FZO-1 in the regulation of mitochondrial morphology. We performed RNAi targeting *fzo-1* either in *vps-13D*(*Δ*) mutant background or in control worms. *fzo-1* knockdown worms exhibited a decrease in average major axis length (17.7 ± 2.5 pixels) and mitochondrial area (77.8 ± 8.5 pixels) compared with control animals, which exhibited a major axis length of 28.4 ± 6.7 pixels and a mitochondrial area of 119.2 ± 9.8 pixels (Fig. 5a–c). Knockdown of *fzo-1* failed to suppress the abnormal mitochondrial morphology observed in *vps-13D*(*Δ*)*-*deficient animals (Fig. 5a–c). The major axis length (20.6 ± 6.5 pixels) and mitochondrial area (146.7 ± 57.4 pixels) of *vps-13D*(*Δ*); *fzo-1*(RNAi) worms were similar to those observed in *vps-13D*(*Δ*) control animals (19.4 ± 4.2 pixels in major axis length and 157.4 ± 40.1 pixels in area). We also examined whether *fzo-1* affects mitochondrial UPR^mt^. Consistent with previous studies (Haeussler *et al*. 2020), the knockdown of *fzo-1* in wild-type worms led to a significant increase in the level of the UPR^mt^ reporter *zcIs13* (Fig. 5d and e). However, *fzo-1* inactivation in *vps-13D*(*Δ*) mutant worms did not result in a significant change in UPR^mt^ compared with the *vps-13D*(*Δ*) single mutant strain (Fig. 5d and e). Collectively, these data suggest that *vps-13D* and *fzo-1* function together to regulate mitochondria homeostasis.

## Discussion

We developed a new model to study *VPS13D*-related SCAR4 disease in the nematode *C. elegans*. Our results indicate that *C. elegans* carrying the *VPS13D* deletions and disease-associated missense mutations, in the worm ortholog *vps-13D*, are viable. However, *vps-13D* null deletion mutations that remove the C-terminus display maternal effect sterility. In addition, *vps-13D* deletions and N3017I missense mutant *C. elegans* exhibit impaired locomotion, thus reflecting a similar role for this gene because human SCAR4 patients exhibit movement deficiencies. Importantly, *vps-13D* mutant *C. elegans* displayed abnormal mitochondrial morphology, and *vps-13D* deletions and *vps-13D(N3017I)* mutants displayed varying levels increased mitochondrial UPR.

The key clinical features of SCAR4 patients include progressive development of hyperkinetic movement disorder (dystonia, chorea, and/or ataxia). SCAR4 patient muscle biopsies exhibited mitochondrial accumulation and mild lipidosis (Gauthier *et al*. 2018). Similarly, altered mitochondrial morphology and mitochondria clearance is decreased in cultured *VPS13D* patient-derived fibroblasts, *VPS13D* mutant human HeLa cells, and *Drosophila* (Anding *et al*. 2018; Seong *et al*. 2018; Shen, Fortier, Zhao, *et al*. 2021). Although mitochondrial respiratory chain function was normal in the muscle biopsy of 1 SCAR4 patient, a decrease in energy production and complex (I, III, and IV) protein levels has been detected in some patient fibroblasts (Gauthier *et al*. 2018; Seong *et al*. 2018; Durand *et al*. 2022). Consistent with the movement defect of SCAR4 patients, worms carrying either deletions or N3017I missense mutations in *vps-13D* exhibited defects in crawling and/or swimming behavior. Interestingly, the abnormal swimming ability of *vps-13D* mutant worms is suggestive of mitochondrial dysfunction since swimming has been shown to be more energetically demanding than crawling for *C. elegans* (Laranjeiro *et al*. 2017; Kang *et al*. 2024). The similarities between SCAR4 patients and *vps-13D* mutant worms, including locomotion defects, mitochondrial morphology, and mitochondrial homeostasis changes, suggest that *C. elegans* will likely be a useful model to reveal other genes in this pathway because of the strength of this genetic model.

Mitochondrial homeostasis is controlled by mitophagy machinery, including the PINK1–Parkin axis (Pickrell and Youle 2015). VPS13D functions downstream of PINK1, but bifurcates with Parkin to regulate mitochondrial clearance (Shen, Fortier, Wang, *et al*. 2021). Specifically, VPS13D binds to ubiquitin and affects both the PINK1 substrate ubiquitinphospho-serine 65 and localization of the mitophagy receptor Ref2p/p62 on mitochondria (Shen, Fortier, Wang, *et al*. 2021). Previous studies suggest mitophagy and mitochondrial UPR are both required to maintain mitochondrial health (Pellegrino and Haynes 2015). Mitophagy and mitochondrial UPR function in a complementary manner to either recycle the damaged mitochondria by autophagy or recover the less damaged mitochondria. Additionally, mitophagy inhibition also causes damaged mitochondrial DNA accumulation that triggers mitochondrial UPR. Recently, it has been shown that nonmitochondrial proteins, including VPS13D, also play a role in maintaining mitochondrial homeostasis (Rolland and Conradt 2022). Although it is unclear whether VPS13D regulates mitochondrial clearance in worms, evidence of VPS13D regulating mitophagy in different animals and human cells suggests this possibility (Anding *et al*. 2018; Shen, Fortier, Wang, *et al*. 2021). Therefore, *vps-13D* mutant worms may potentially accumulate damaged mitochondria to activate mitochondrial UPR.

Mitochondria and ER contact dictates interorganelle lipid transfer sites and mitochondrial membrane lipid composition (Gatta and Levine 2017). The lipid composition of mitochondrial membranes, in turn, can influence crucial processes within mitochondria (Böckler and Westermann 2014; Lahiri *et al*. 2015). Previous studies indicate Vmp1 and Vps13D regulate mitochondria–ER contact and mitophagy (Shen, Fortier, Zhao, *et al*. 2021). VPS13D binds the outer mitochondrial membrane GTPase-Miro (both Miro1 and Miro2), likely via the WD40-like/VAB domain (Guillén-Samander *et al*. 2021), and the conserved C-terminal regions have been shown to work synergistically with the VAB domain to facilitate the membrane targeting (Dziurdzik and Conibear 2021). Moreover, the VPS13D C-terminus is similar to the lipid transfer protein ATG2 (Öztop-Çakmak *et al*. 2022). Consistent with the importance of these domains, our data indicate that *vps-13D* C-terminus is important for mitochondrial homeostasis.

The mitochondrial protein MFN2 facilitates mitochondrial fusion, as well as mitochondria and ER contact (Filadi *et al*. 2018). Mutations in *MFN2* are associated with Charcot–Marie–Tooth disease type 2A, in which mitochondrial morphology is abnormal in nerve tissue and fibroblasts from patients (Verhoeven *et al*. 2006; Amiott *et al*. 2008). Similarly, loss of *fzo-1*, the worm *MFN2* homolog, resulted in progressive movement deficits, fragmented mitochondria, and mitochondrial UPR activation in *C. elegans* (Byrne *et al*. 2019; Haeussler *et al*. 2021). Consistent with these studies, our results indicate *fzo-1* knockdown worms displayed altered mitochondrial morphology and induced mitochondrial UPR. UPR^mt^ activation requires the key transcription factor ATFS-1, which regulates the *hsp-6* reporter expression (Nargund *et al*. 2015; Lin *et al*. 2016; Shpilka *et al*. 2021; Xin *et al*. 2022). Notably, knockdown of *atfs-1* significantly suppressed UPR^mt^ in the *fzo-1* mutant worms, confirming that UPR^mt^ induction by loss of *fzo-1* is ATFS-1 dependent (Chen *et al*. 2021). Unlike *Marf/MFN2* function in the Vps13D pathway regulating mitochondrial morphology in *Drosophila* (Shen, Fortier, Zhao, *et al*. 2021), our results suggest that *fzo-1* and *vps-13D* could function in a related pathway to affect mitochondrial UPR in *C elegans*. In summary, this study provides evidence supporting the conserved role of VPS13D in regulating mitochondrial morphology and function in *C. elegans* and provides insights into how disease mutations affect mitochondrial homeostasis and behavior.

## Supplementary Material

jkaf023_Supplementary_Data

## Data Availability

All data files from this study have been uploaded to Mendeley Data (DOI: 10.17632/3mwgvd2dbx.1). Supplemental material available at G3 online.
