# Supplementary material for: VPS13D mutations affect mitochondrial homeostasis and locomotion in Caenorhabditis elegans
Source: G3 (Bethesda). 2025 Feb 17;15(4):jkaf023. doi: 10.1093/g3journal/jkaf023 (PMC12005150; doi:10.1093/g3journal/jkaf023)
Supplement: jkaf023_Supplementary_Data [file jkaf023_supplementary_data.zip › Figure_S1_Legend_G3-2025-405672.docx]

**Supplemental Figure Legend**

**Figure S1.** Day 1 *vps-13D* mutant worms did not show a significant deficiency in locomotion.

(a) Comparison of average velocity for 1-day-old young adult wild type and *vps-13D* mutant animals from the final 5 mins MWT recordings. The velocity has been normalized and calculated based on the worm body lengths of respective strains. The difference in average velocity between wild-type and mutants was analyzed by one-way ANOVA with Dunnett’s multiple comparison test.

(b) Number of thrashes per minute in M9 liquid for 1-day-old young adult wild type and *vps-13D* mutant animals (n=10-15). Differences between wild type and mutants were analyzed by One-way ANOVA with Dunnett correction for multiple comparisons.
