# Supplementary material for: VPS13D mutations affect mitochondrial homeostasis and locomotion in Caenorhabditis elegans
Source: G3 (Bethesda). 2025 Feb 17;15(4):jkaf023. doi: 10.1093/g3journal/jkaf023 (PMC12005150; doi:10.1093/g3journal/jkaf023)
Supplement: jkaf023_Supplementary_Data [file jkaf023_supplementary_data.zip › Tables_S2-S4_G3-2025-405672.docx]

Supplemental Tables S2-S4

**Table S2: crRNA (s)** (purchased from IDT)

| **crRNA** | **Sequence (5´to 3´)** |
| --- | --- |
| crRNA (*zf194*) (*N2454S*) | CTTTCTTGTTGATAAAGTAC |
| crRNA (*zf195*) (*N3017I*) | GTCAAACAGTGTCTCAAAAG |
| crRNA (*zf196*) (*R3144Q*) | AGAGACGAGGGATTCGTACA |
| crRNA (*zf197*) (*ΔC*) | 1. CTTTCTTGTTGATAAAGTAC 2. AGAGACGAGGGATTCGTACA |

**Table S3: HR template (s)** (purchased from IDT)

| **HR template** | **Sequence (5´to 3´)** |
| --- | --- |
| HR template (*zf194*) (*N2454S*) | ATGCCGATTTGTATCCACCACCAATCAGAATTGAAAGCTTGACTGATGTACCGGTACTTTATCAACAAGAAAGTAGTACTCCTGC |
| HR template (*zf195*) (*N3017I*) | AGAGATTTGTTGCTGGACTTGGATATGGATTGTCAATCTCGGTCTCAAAAGTCGCATCATCCATGGCTTCGGGTGTCGGTGCA |
| HR template (*zf196*) (*R3144Q*) | TTTATTTTCAAATCTATCCAATCAAAAATTTCAGCCAGGTTCGTATCCCGCGGCTCTGTCGAAACCTGTACCACC |
| HR template (*zf197*)  (*ΔC*) | TACAGGTTTCGACAGAGACGAGGGATTCGTACTCACGGAACATCCGTCAAATTTTCAATTCTGATTGGTG |

**Table S4: DNA primer (s)**

| **Primer** | **Sequence (5´to 3´)** |
| --- | --- |
| (*zf194*) (*N2454S*) genotyping F | GCTGATGTCAAACACTGGAG |
| (*zf194*) (*N2454S*) genotyping R | CTTCCGTTCTACACTGCTTGT |
| (*zf195*) (*N3017I*) genotyping F | AGCACTCAACTTACCACCGA |
| (*zf195*) (*N3017I*) genotyping R | GACACCTGTAGCTGGCATTG |
| (*zf196*) (*R3144Q*) genotyping F | GTTGCGACTGGTGTTGTTGA |
| (*zf196*) (*R3144Q*) genotyping R | GCGTGCCACTTCAATTCTACT |
| (*zf197*) (*ΔC*) genotyping F1 (external primer) | AGAACACAAGACCAAACCGAC |
| (*zf197*) (*ΔC*) genotyping R1 | TGAGAAGCTCCATCTGAGCA |
| (*zf197*) (*ΔC*) genotyping F2 (internal primer) | TTACCCCCATTCCGACAGTA |
| (*ok2632*) (*Δ*) genotyping F1 (external primer) | GGAATTCGAAAACCACGATG |
| (*ok2632*) (*Δ*) genotyping R1 (external primer) | GATGAGATTTCGATTGCCGAG |
| (*ok2632*) (*Δ*) genotyping F2 (internal primer) | GTTCTTTATGAGGCCCGACA |
| (*ok2632*) (*Δ*) genotyping R2 (internal primer) | GCCAGAGCATTTGATGGATT |
