# Supplementary material for: VPS13D mutations affect mitochondrial homeostasis and locomotion in Caenorhabditis elegans
Source: G3 (Bethesda). 2025 Feb 17;15(4):jkaf023. doi: 10.1093/g3journal/jkaf023 (PMC12005150; doi:10.1093/g3journal/jkaf023)
Supplement: jkaf023_Supplementary_Data [file jkaf023_supplementary_data.zip › Table_S1_G3-2025-405672.pdf]

Supplemental Tables S1

Table S1: List of strains used in this study.

Strain	Genotype
N2	Wild type
VC1998	*C25H3.11* (*ok2632*)/*mIn1* [*mIs14 dpy-10* (*e128*)] II.
QW2428	*vsp-13D* (*zf194*) (*N2454S*) II.
QW2451	*vsp-13D* (*zf195*) (*N3017I*) II.
QW2453	*vsp-13D* (*zf196*) (*R3144Q*) II.
QW2526	*vsp-13D* (*zf197*) (*ΔC*)/ *mIn1* [*mIs14 dpy-10* (*e128*)] II.
QW2540	*lin-15* (*n765ts*); *zfEx1225* [P $_{vsp\text{-}13D}$::GFP; *lin-15* (+)].
SJ4103	*zcIs14* [*myo-3*::GFP (*mit*)].
QW2563	*vsp-13D* (*zf194*) (*N2454S*) II; *zcIs14* [*myo-3*::GFP (*mit*)].
QW2564	*vsp-13D* (*zf195*) (*N3017I*) II; *zcIs14* [*myo-3*::GFP (*mit*)].
QW2565	*vsp-13D* (*zf196*) (*R3144Q*) II; *zcIs14* [*myo-3*::GFP (*mit*)].
QW2566	*C25H3.11* (*ok2632*)/*mIn1* [*mIs14 dpy-10* (*e128*)] II; *zcIs14* [*myo-3*::GFP (*mit*)].
QW2567	*vsp-13D* (*zf197*) (*ΔC*)/ *mIn1* [*mIs14 dpy-10* (*e128*)] *II*; *zcIs14* [*myo-3*::GFP (*mit*)].
GL347	*zcIs13* [*hsp-6*p::GFP + *lin-15* (+)] V.
QW2553	*vsp-13D* (*zf194*) (*N2454S*) II; *zcIs13* [*hsp-6*p::GFP + *lin-15* (+)] V.
QW2491	*vsp-13D* (*zf195*) (*N3017I*) II; *zcIs13* [*hsp-6*p::GFP + *lin-15* (+)] V.
QW2527	*vsp-13D* (*zf196*) (*R3144Q*) II; *zcIs13* [*hsp-6*p::GFP + *lin-15* (+)] V.
