# Supplementary material for: VPS13D mutations affect mitochondrial homeostasis and locomotion in Caenorhabditis elegans
Source: G3 (Bethesda). 2025 Feb 17;15(4):jkaf023. doi: 10.1093/g3journal/jkaf023 (PMC12005150; doi:10.1093/g3journal/jkaf023)
Supplement: jkaf023_Supplementary_Data [file jkaf023_supplementary_data.zip › Table_S5_G3-2025-405672.docx]

Supplemental Tables S5

**Table S5: VPS13D protein homology with related human, *C. elegans* and *Drosophila melanogaster* proteins.**

| Species | Protein | Gene | Length | Identity | Similarity | Gaps |
| --- | --- | --- | --- | --- | --- | --- |
| *Homo sapiens* | VPS13D | *VPS13D* | 4388  (NP_056193.2) | - | - | - |
|  | VPS13A | *VPS13A* | 3174  (NP_150648.2) | 17% | 32% | 39% |
|  | VPS13B | *VPS13B* | 4022  (NP_060360.3) | 18% | 31% | 36% |
|  | VPS13C | *VPS13C* | 3753  (NP_065872.1) | 20% | 35% | 28% |
| *C. elegans* | VPS-13D | *vps-13D*  (*C25H3.11*) | 3314  (NP_001348703.1) | 22% | 36% | 36% |
|  | VPS-13A | *vps-13A* (*T08G11.1*) | 3212  (NP_740900.1) | 18% | 32% | 36% |
| *D. melanogaster* | Vps13D | *Vps13D* | 3919  (NP_729825.2) | 29% | 47% | 17% |
