# Supplementary figures and images for: VPS13D mutations affect mitochondrial homeostasis and locomotion in Caenorhabditis elegans
Source: G3 (Bethesda). 2025 Feb 17;15(4):jkaf023. doi: 10.1093/g3journal/jkaf023 (PMC12005150; doi:10.1093/g3journal/jkaf023)

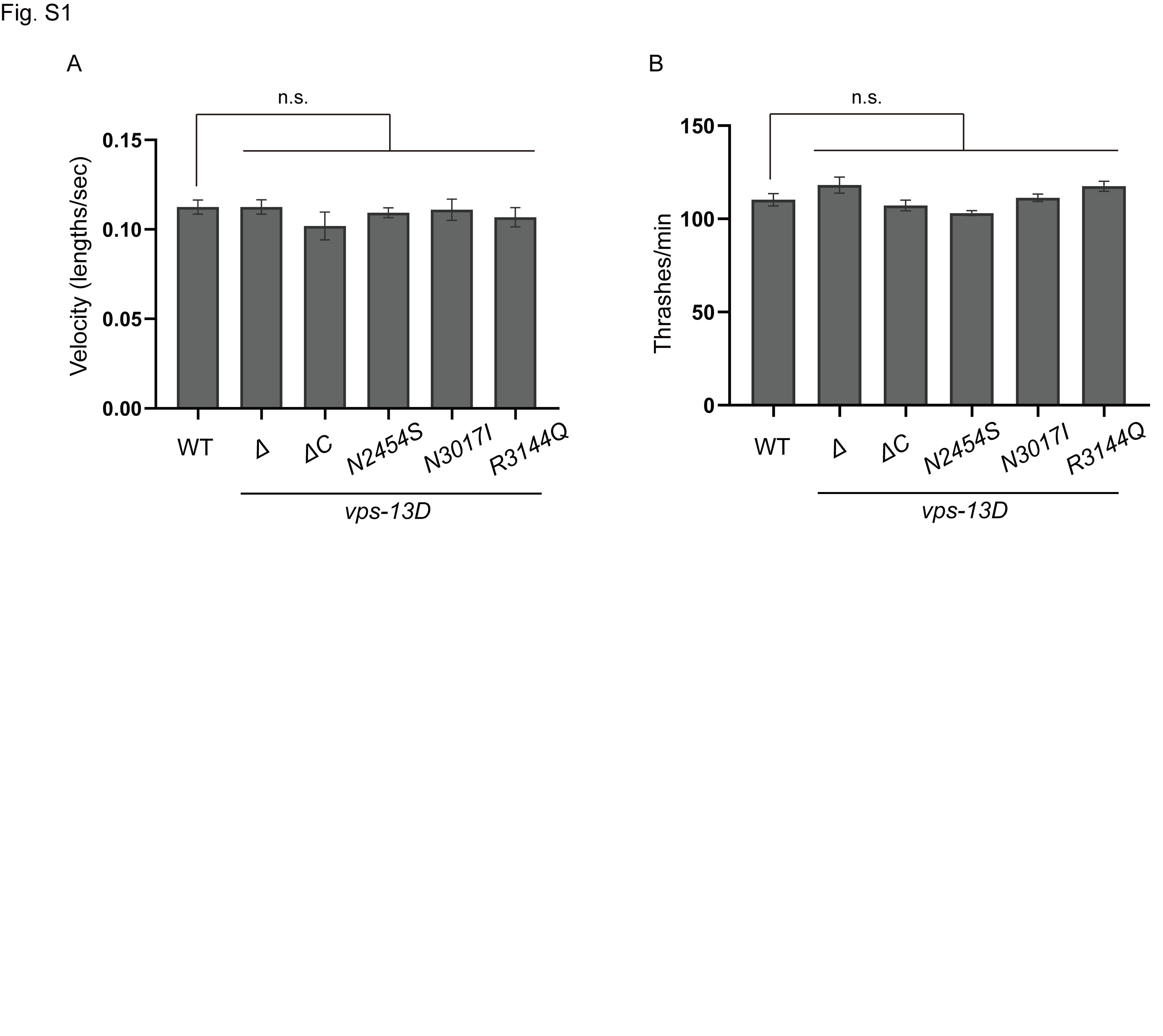

Supplement: jkaf023_Supplementary_Data [file jkaf023_supplementary_data.zip › Figure_S1_G3-2025-405672.tif]
